# Comparison of the Effects of Intraoperative Dexmedetomidine and Fentanyl Infusion on Postoperative Agitation and Analgesia in Pediatric Patients Undergoing Tonsillectomy and Adenoidectomy: A Prospective Randomized Trial

**DOI:** 10.3390/children13050700

**Published:** 2026-05-20

**Authors:** Yasar Gokhan Gul, Sümeyye Yildiz, Hande Güngör, Burak Omur, Pelin Karaaslan, Bahadir Ciftci

**Affiliations:** 1 Department of Anesthesiology and Reanimation, Istanbul Medipol University, Istanbul 34214, Turkey; yasar.gul@medipol.edu.tr (Y.G.G.); sumeyye.yildiz@medipol.edu.tr (S.Y.); hande.gungor@medipol.edu.tr (H.G.); bomur@medipol.edu.tr (B.O.); pkaraaslan@medipol.edu.tr (P.K.); 2 Department of Anatomy, Istanbul Medipol University, Istanbul 34726, Turkey

**Keywords:** dexmedetomidine, fentanyl, agitation, FLACC, pediatric anesthesia

## Abstract

**Highlights:**

**What are the main findings?**

• Intraoperative dexmedetomidine infusion provides significantly superior analgesia compared to fentanyl in pediatric patients undergoing tonsillectomy and adenoidectomy, particularly at the 30-min postoperative mark.
• The use of dexmedetomidine, compared to traditional fentanyl administration, achieves high-quality early postoperative pain control without prolonging extubation times or causing hemodynamic instability.

**What are the implications of the main findings?**

• Dexmedetomidine serves as a potent and safe opioid-sparing alternative for pediatric airway surgeries, offering more stable analgesia during the critical early recovery “transition period”.
• These results support the clinical integration of alpha-2 agonists to enhance postoperative comfort and recovery quality in children while avoiding the respiratory risks associated with conventional opioids.

**Abstract:**

**Background/Objectives:** Postoperative agitation (PA) and postoperative pain in pediatric patients following sevoflurane anesthesia are challenging clinical scenarios. This study aimed to evaluate the effects of intraoperative dexmedetomidine infusion compared to fentanyl infusion on the prevention of postoperative agitation and analgesic efficacy in children undergoing tonsillectomy and/or adenoidectomy. **Methods:** After ethical committee approval, a total of 85 pediatric patients (age range: 2–13 years) in the ASA I-II group were included in the study. Patients were randomized into two groups: the dexmedetomidine group (Group D, n = 40) and the fentanyl group (Group F, n = 45). Postoperative pain was monitored in the recovery unit (PACU) using the FLACC (face, legs, activity, cry, consolability) scale, and agitation was monitored using the PAED (pediatric anesthesia emergence delirium) scale. FLACC and PAED were monitored at 5, 10, 15, 30 min, and 2 and 4 h postoperatively. **Results:** Demographic data and surgical durations were similar between groups (*p* > 0.05). The dexmedetomidine group had lower FLACC pain scores at 10 and 15 min (uncorrected trends), but only the difference at 30 min remained statistically significant after Bonferroni correction (*p* = 0.0001; Cohen’s d = 0.85). Although PAED scores were numerically lower in Group D, no statistically significant difference was found. While an observational trend toward lower agitation was noted, it did not reach statistical significance. Extubation times and hemodynamic parameters were similar in both groups. **Conclusions:** The intraoperative use of dexmedetomidine in tonsillectomy and adenoidectomy procedures provides superior analgesia compared to fentanyl, particularly in the first 30 min postoperatively, without prolonging recovery time.

## 1. Introduction

Tonsillectomy and adenoidectomy are among the most frequently performed surgical procedures in the pediatric population [1]. Although sevoflurane is the common inhalation agent in these operations, it may reach emergence agitation (EA) in pediatric patients at a rate of 40% [2]. EA is characterized by crying, restlessness, disorientation, and risk of self-harm, and can lead to problems such as surgical site bleeding, catheter dislodgement, and parental dissatisfaction [3].

Various agents such as opioids (fentanyl, etc.), alpha-2 agonists, and ketamine are used to prevent EA and manage postoperative pain. Although fentanyl is a potent analgesic, it carries risks of side effects such as respiratory depression, nausea and vomiting, and prolonged sedation [4]. Dexmedetomidine, a selective alpha-2 adrenergic agonist, stands out for providing sedative, anxiolytic, and analgesic effects without causing respiratory depression [3]. Although studies in the literature show that dexmedetomidine reduces postoperative agitation [5,6], those focusing on the power of its analgesic efficacy over time compared to routine fentanyl use are limited.

Combining dexmedetomidine with other non-opioid drugs as part of an opioid-sparing strategy may provide a safer and more efficient way to manage pain while lowering the risk of respiratory depression and emerging agitation. This study aimed to compare the effects of intraoperative dexmedetomidine infusion versus traditional fentanyl administration on postoperative agitation (PAED) and pain (FLACC) scores in pediatric patients undergoing tonsillectomy and adenoidectomy. Our study hypothesizes that intraoperative dexmedetomidine infusion, compared to fentanyl, will reduce postoperative agitation and pain scores without clinically significantly prolonging the recovery time.

## 2. Materials and Methods

### 2.1. Study Design and Patient Selection

This study was conducted prospectively, randomly, and in a controlled manner after obtaining approval from the Ethics Committee of Istanbul Medipol University (date: 22 June 2023; decision no.: 530) and is registered at Clinicaltrials.gov (NCT05986942). After describing the study’s methodology to the parents of each child, we received their written informed consent. The study included 85 children, with an age range of 2–13 years, with ASA physical status I-II who were scheduled for elective tonsillectomy and/or adenoidectomy. Exclusion criteria included an ASA status ≥ 4, known hypersensitivity or allergy to acetaminophen, dexmedetomidine, or fentanyl, chronic opioid therapy, renal or hepatic disease, neurological disorders, children anticipated to experience airway difficulties, and obesity (BMI ≥ 99th percentile for their age). The study was conducted at Medipol Mega University Hospital between October and November 2023, in accordance with the principles of the Declaration of Helsinki.

### 2.2. Randomization and Blinding

Patients were randomized into two equal groups using a computer-generated random number sequence. Allocation concealment was maintained via opaque, sequentially numbered, sealed envelopes. The sequence generation and allocation assignment were managed by an independent research assistant not involved in data collection or clinical care. The study maintained blinded design; patients, parents, surgeons, and the PACU nurse anesthetists who collected the postoperative outcome data were blinded to group allocation. The attending anesthesiologists in the operating room were not blinded as they prepared and administered the study drugs. Additionally, the statistical analyst was blinded to the group assignments to ensure unbiased evaluation.

### 2.3. Anesthesia Management

Oral midazolam (0.5 mg/kg to a maximum dose of 20 mg) was given to all patients as premedication around half an hour before induction. Upon arrival in the operating room, standard monitoring was applied to all patients, including electrocardiography (ECG), non-invasive blood pressure (NIBP), peripheral oxygen saturation (SpO_2_), and end-tidal carbon dioxide (EtCO_2_). These parameters were monitored continuously throughout the procedure to ensure hemodynamic and respiratory stability. A peripheral intravenous catheter was inserted after sevoflurane in oxygen was used to induce anesthesia. Participants were divided into two groups at random: Group D received propofol (2 mg/kg) + rocuronium (0.6 mg/kg) and dexmedetomidine, while Group F received propofol (2 mg/kg) + rocuronium (0.6 mg/kg) and fentanyl (1 µg/kg). In Group D, following a 2 mcg/kg loading dose administered over 10 min, an IV dexmedetomidine infusion at a rate of 0.7 mcg/kg/hour was administered until the last 5 min of the operation. Sevoflurane in an oxygen and air mixture was used for maintenance anesthesia, while ondansetron (0.15 mg/kg) and dexamethasone (0.5 mg/kg) were administered to avoid postoperative nausea and vomiting; all patients received intravenous acetaminophen (10 mg/kg) during the perioperative period. Normal saline at a volume of 10–20 mL/kg was used for intraoperative fluid management. Patients were extubated after spontaneous breathing signs were observed following reversal of the effects of muscle relaxants with 2 mg/kg IV sugammadex and were transferred to the recovery unit. The time from the end of surgery to tracheal extubation was recorded as the extubation time.

### 2.4. Postoperative Period

Following surgery, the patients were sent to the recovery area, where the FLACC (face, legs, activity, cry, consolability) scale was used to measure their level of pain until they were released. Recovery was defined as achieving an Aldrete score of ≥8. The pediatric anesthesia emergence delirium (PAED) scale was used in the recovery room to assess emergence delirium. Patients remained in the post-anesthesia care unit (PACU) for an average of 45 min and were transferred to the clinical ward once discharge criteria were met, including stable vital signs and the capacity to continue oral intake [6,7]. FLACC and agitation PAED assessments at 2 and 4 h were conducted in the clinical ward setting.

In our study, intravenous paracetamol was utilized as the standard postoperative analgesic. This choice reflects our institution’s established clinical protocol for pediatric surgical procedures, where paracetamol is the preferred agent for early postoperative pain management. While the addition of NSAIDs is a recognized component of multimodal analgesia, our study aimed to compare the specific adjuvant effects of dexmedetomidine and fentanyl within the framework of our existing clinical standards.

### 2.5. Data Collection and Evaluation of Outcomes

This study aimed to evaluate and compare the effects of intraoperative dexmedetomidine infusion versus fentanyl infusion on both postoperative agitation and analgesic efficacy in children undergoing tonsillectomy and/or adenoidectomy.

Pain and agitation assessments were performed using the FLACC and PAED scales at 5, 10, 15, and 30 min in the PACU, and subsequently at 2 and 4 h in the clinical ward setting.

The primary outcome of the study was the FLACC scores at 30 min postoperatively.

Pain Assessment: This was performed using the FLACC (face, legs, activity, cry, consolability) scale at 5, 10, 15, and 30 min and at 2 and 4 h postoperatively.

Agitation Assessment: This was performed using the PAED (pediatric anesthesia emergence delirium) scale at the same time intervals.

Hemodynamic Data: Heart rate and oxygen saturation values were recorded.

The outcomes were evaluated by a nurse anesthetist who was blinded to the procedure.

### 2.6. Power Analysis

The sample size was determined a priori using a power analysis based on a preliminary pilot study of 16 patients (n = 8 per group) randomly selected from the study population. In this pilot cohort, the mean postoperative 30-min FLACC scores were 2.00 ± 0.93 for Group D and 3.62 ± 2.92 for Group F, representing an effect size (Cohen’s d) of 0.749. With a significance level (α) of 0.05 and a power (1-β) of 0.80, the minimum required sample size was calculated as 29 patients per group (G*Power 3.1.9.7). To account for potential dropouts and strengthen the analysis of secondary outcomes, we enrolled a total of 85 patients in the final trial.

### 2.7. Statistical Analysis

Statistical analysis and calculations of the data were performed using statistical software (SPSS version 22.0; IBM Corporation, Armonk, NY, USA), and the normality of data distribution was assessed using the Kolmogorov–Smirnov test. Numerical data are presented as arithmetic mean ± standard deviation (Mean ± SD) and median (minimum–maximum) values for data not following a normal distribution, while categorical data are expressed as numbers and percentages (%). To compare two groups, the Mann–Whitney U test was used for numerical variables that did not show a normal distribution and for ordinal data-type scores (FLACC and PAED scores). The Chi-square test was used for the comparison of categorical variables between groups. The level of statistical significance was accepted as *p* < 0.05. Due to repeated measurements across 6 different time points for FLACC and PAED scores, a Bonferroni correction was applied to adjust for multiplicity; thus, a *p*-value < 0.0083 (0.05/6) was considered statistically significant for these outcomes.

## 3. Results

**Figure 1:**
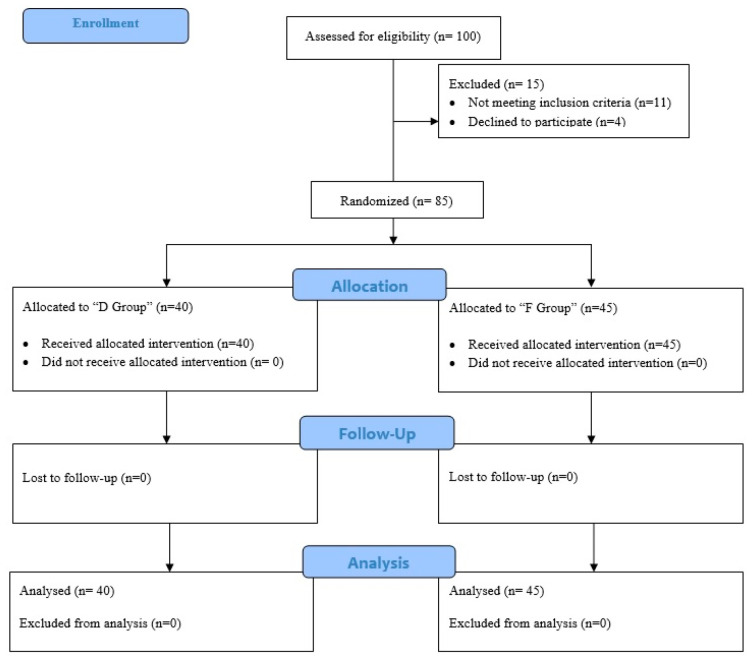
CONSORT flow diagram of the study.

In total, 100 children were assessed, of which 85 were included in the study (CONSORT—Figure 1), with 40 patients in Group D and 45 patients in Group F. There was no statistically significant difference between the groups in terms of age, weight, height, gender distribution, anesthesia duration, and surgical duration (*p* > 0.05) (Table 1).

*Extubation and Recovery:* The median time from sevoflurane withdrawal to extubation was 14.85 ± 6.38 min in Group D and 13.71 ± 7.56 min in Group F. This difference was not statistically significant (*p* = 0.194), and dexmedetomidine did not delay recovery.

*Analgesic Effect (FLACC Scores):* The dexmedetomidine group showed lower pain scores in the early postoperative period compared to the fentanyl group. Analgesic efficacy was evaluated across six time points; while Group D showed superior results early on, the difference in FLACC scores between the groups was not significant at the 2-h and 4-h marks (*p* = 0.2 and *p* = 0.6, respectively).

Tenth and Fifteenth Minute: The median FLACC score was 1 in Group D and 2 in Group F for both time points. While these values showed a downward clinical trend (uncorrected *p* = 0.049 and *p* = 0.045, respectively), they did not meet the stringent Bonferroni-adjusted significance threshold.

Thirtieth Minute: The most significant difference was observed during this time interval. The median FLACC score was 1 (0–5) in Group D and 3 (1–9) in Group F (*p* < 0.001). This difference was highly statistically significant even after Bonferroni correction (*p* = 0.0001).

The difference in analgesic efficacy disappeared after the second hour (Table 2).

*Agitation (PAED Scores):* At all measurement times (5, 10, 15, and 30 min), the median PAED scores were numerically lower or equivalent in the dexmedetomidine group compared to the fentanyl group. However, these differences did not reach statistical significance at any time point (*p* > 0.05) (Table 3).

There was no significant difference in terms of heart rate and oxygen saturation values between groups (Table 4).

All patients received intravenous acetaminophen (10 mg/kg) perioperatively. No rescue opioids or additional analgesics were required in the PACU or during the postoperative follow-up period in either group. Furthermore, no respiratory complications, such as laryngospasm or significant desaturation (SpO_2_ < 90), were observed during or after extubation.

## 4. Discussion

This study demonstrated that intraoperative dexmedetomidine infusion in children undergoing tonsillectomy and adenoidectomy provided superior analgesia compared to fentanyl, particularly during the first 30 min postoperatively, without prolonging recovery time. The most notable finding of our study was the significant difference in FLACC scores at the 30-min mark. Our power analysis showed that the effect in this time frame was “large” (Cohen’s d = 0.85) and that the study reached 97% power, proving that dexmedetomidine provides more stable and potent pain control than fentanyl in the early postoperative period.

According to a scoping review protocol, dexmedetomidine is such an effective and reliable agent for preventing postoperative agitation in pediatric tonsillectomy and adenoidectomy surgeries that it has become a reference drug against which newly emerging agents are compared in current research [3]. Indeed, in a recently published randomized controlled trial by Liu et al. (2025), dexmedetomidine was selected as the comparison group while investigating the efficacy of remimazolam, a new benzodiazepine derivative [7]. Liu et al. confirmed the success of dexmedetomidine in maintaining hemodynamic stability and improving recovery quality in this surgical group, while also supporting its role in agitation management with current data. In our study, comparing dexmedetomidine with fentanyl, the most commonly used opioid in a clinical setting, and demonstrating its undisputed analgesic superiority (Cohen’s d = 0.85) especially at 30 min postoperatively, complements the findings of Liu and colleagues. In our study, while the PAED scores were numerically lower in the dexmedetomidine group, this difference did not reach statistical significance. This observational trend aligns generally with the literature, but we were unable to establish a definitive statistical advantage for agitation in this specific cohort.

In pediatric tonsillectomy cases, fentanyl is often a reliable option compared to other agents in the search for the ideal adjuvant agent. According to a study comparing ketamine and fentanyl, the incidence of agitation upon awakening was significantly lower in the fentanyl group (3.57%) compared to the ketamine group (19.35%), and fentanyl was reported to be superior to ketamine in terms of recovery quality [8]. Considering the success of fentanyl over ketamine, it is noteworthy that in our study, dexmedetomidine provided significantly superior analgesia (significant decrease in FLACC scores) even compared to fentanyl, an agent with proven efficacy, particularly at 30 min postoperatively. We demonstrated that dexmedetomidine provided much more stable pain control at the critical ‘transition period’ 30 min postoperatively.

Sultana et al. (2022), in their study of children undergoing surgery under sevoflurane anesthesia, reported that the incidence of agitation upon awakening was significantly lower in the dexmedetomidine group compared to the fentanyl group [9]. Although the PAED scores in our study did not reach statistical significance, the fact that the mean scores were clinically lower in the dexmedetomidine group is consistent with the findings of Sultana et al. However, the most important point that distinguishes our study from theirs and contributes uniquely to the literature is the time distribution of analgesic efficacy beyond agitation. While they focused on the incidence of general agitation, we demonstrated that dexmedetomidine provided much more stable pain control (superiority proven with 97% statistical power in FLACC scores) at the critical ‘transition period’ 30 min postoperatively, when the analgesic effect of fentanyl began to wane. This finding demonstrates that dexmedetomidine not only prevents agitation (sedative effect) but also directly impacts postoperative comfort as a potent analgesic agent that reduces opioid consumption.

In our study, it was observed that hemodynamic parameters, specifically heart rate, remained stable in the dexmedetomidine group and did not differ negatively from the fentanyl group. This safety profile is also supported by a recent study by Cheng et al. (2025) in children undergoing cochlear implantation [10]. Cheng et al. reported that hemodynamic parameters were more consistent in children receiving dexmedetomidine infusion compared to the control group and that agitation and crying scores were significantly lower. The results of our study are consistent with the findings of Cheng et al., confirming that dexmedetomidine provides safe and comfortable recovery without compromising cardiovascular stability in pediatric patients, even in different types of surgery.

The efficacy of dexmedetomidine in postoperative delirium and pain management has been demonstrated not only in otolaryngological surgeries but also in more invasive procedures. In a retrospective analysis by Sun et al. (2025) comparing dexmedetomidine with the potent opioid sufentanil in children undergoing lower extremity orthopedic surgery, the incidence of postoperative delirium in the dexmedetomidine group (14.1%) was significantly lower than that in the sufentanil group (32.8%) [11]. Sun et al. also noted that postoperative FLACC scores were lower in the dexmedetomidine group and that the need for additional analgesics was reduced. In our study, the strong analgesic efficacy achieved with dexmedetomidine compared to the fentanyl group and the downward trend in agitation scores are consistent with the findings of Sun et al. in orthopedic surgery. This situation provides strong evidence that dexmedetomidine provides more stable neurocognitive recovery and analgesia compared to opioids, regardless of the type of surgery.

A critical consideration in the use of dexmedetomidine is whether its sedative properties mask postoperative pain scores [12,13,14]. However, in our study, PAED scores—which reflect delirium and awareness levels—did not differ significantly between groups. This suggests that Group D patients were not more deeply sedated than Group F patients, supporting the conclusion that the lower FLACC scores represent a genuine analgesic benefit rather than a side effect of sedation [6,7]. A valid concern in studies involving alpha-2 agonists is whether lower pain scores reflect true analgesia or are simply a result of drug-induced somnolence. In our study, several factors suggest a genuine analgesic benefit. First, extubation times and the time to reach an Aldrete score of ≥8 were comparable between the dexmedetomidine and fentanyl groups, suggesting that Group D patients were not excessively sedated. Second, PAED scores showed a similar level of cognitive emergence in both groups, rather than deep sedation. However, it is possible that the sedative properties of dexmedetomidine contributed to the overall comfort and lower FLACC scores during the critical 30-min transition period, even if it did not delay clinical recovery.

This study has some limitations. End-tidal sevoflurane concentrations at the time of extubation and minor supplemental doses of propofol administered at the discretion of the attending anesthesiologist were not systematically recorded. Additionally, while PACU discharge was standardized by Aldrete scores, specific stay durations were not recorded as a primary outcome variable. Furthermore, while non-invasive blood pressure (NIBP) was monitored for clinical safety, these values were not systematically recorded for statistical analysis. Therefore, our conclusions regarding hemodynamic stability are limited to the heart rate and oxygen saturation data analyzed.

## 5. Conclusions

The use of dexmedetomidine in pediatric tonsillectomy and adenoidectomy procedures provides more effective early postoperative analgesia compared to fentanyl, without prolonging extubation time or causing hemodynamic instability. While there was a clinical trend towards lower agitation scores, this did not reach statistical significance. Overall, dexmedetomidine represents a safe and potent alternative to fentanyl in pediatric airway surgeries.

## Figures and Tables

**Table 1:** Comparison of demographic data and duration times of surgery and anesthesia.

	Group D n: 40	Group F n: 45	*p*
**Age**	6 (3–13)	6 (2–12)	0.7
**Height (cm)**	117.5 (108.2–126.2)	115.0 (110.0–127.0)	0.5
**Weight (kg)**	21.0 (16.0–24.5)	21.0 (17.8–26.5)	0.6
**Duration of Surgery (min)**	62 (25–100)	70 (38–105)	0.1
**Duration of Anesthesia (min)**	40 (10–90)	52 (20–85)	0.06
**Extubation Time (min)**	14 (5–30)	10 (4–35)	0.1

Values are expressed as median (25th–75th percentiles) or number. *p* value is obtained using the Mann–Whitney U test. *p* value is obtained using Pearson’s χ2 test (n). cm: centimeter; kg: kilogram; min: minutes.

**Table 2:** Comparisons of FLACC scores between groups.

	Group D n:40	Group F n:45	*p*
5th min	1 (0–8)	2 (0–10)	0.3
10th min	1 (0–8)	2 (0–6)	**0.04**
15th min	1 (0–8)	2 (0–7)	**0.04**
30th min	1 (0–5)	3 (1–9)	**0.0001**
2nd hour	2 (0–8)	2 (0–10)	0.2
4th hour	2 (0–6)	2 (0–8)	0.6

Data are expressed as median (25th–75th percentiles). *p* value is obtained using the Mann–Whitney U test median (25th–75th percentiles). *p* values are bolded values are statistically significant.

**Table 3:** Comparisons of PAED scores between groups.

	Group D n: 40	Group F n: 45	*p*
5th min	4 (1–20)	4 (0–20)	0.3
10th min	4 (0–18)	4 (0–20)	0.2
15th min	3 (0–15)	4 (0–16)	0.4
30th min	2 (0–10)	2 (0–18)	0.4
2nd hour	2 (0–8)	2 (0–10)	0.1
4th hour	2 (0–8)	2 (0–8)	0.8

Data are expressed as median (25th–75th percentiles). *p* value is obtained using the Mann–Whitney U test for median (25th–75th percentiles).

**Table 4:** Comparisons of postoperative hemodynamic values between groups.

	Group D n:40	Group F n:45	*p*
**Heart rate** at 5th min **(beats/min)**	110 (70–165)	111 (75–165)	0.6
Heart rate at 15th min	106 (69–150)	110 (76–163)	0.7
Heart rate at 30th min	100 (75–132)	98 (75–139)	0.5
**Oxygen saturation** at 5th min **(%)**	98 (92–100)	97 (89–100)	0.3
Oxygen saturation at 15th min	99 (96–100)	98 (92–100)	0.1
Oxygen saturation at 30th min	99 (96–100)	98 (96–100)	0.9

Data are expressed as median (25th–75th percentiles). *p* value is obtained using the Mann–Whitney U test for median (25th–75th percentiles).

## Data Availability

The datasets generated and analyzed during the current study are available from the corresponding author upon reasonable request.

## Abbreviations

The following abbreviations are used in this manuscript:

ASA	American Society of Anesthesiologists
BMI	Body Mass Index
EA	Emergence Agitation
FLACC	Face, Legs, Activity, Cry, Consolability
IV	Intravenous
PA	Postoperative Agitation
PACU	Post-Anesthesia Care Unit
PAED	Pediatric Anesthesia Emergence Delirium
